# Modern Sociology

**Published:** 1899-09-09

**Authors:** 


### Modern Sociology.

### THE ETHICS OF SUICIDE.—II.

By Francis Scougall.

I dealt in my last article with the subject of suicide in its relation to the uneducated classes of our population, and dwelt briefly on their total disbelief in a future state or a Supreme Judge, which, with the great majority, is the main factor in their recourse to self-destruction. But there is another negative influence which acts almost as powerfully on their limited mental consciousness. They refuse absolutely to admit that "the Everlasting has fixed His canon 'gainst self-slaughter," and that the murder of themselves is as distinct a crime as the slaying of a fellow-creature. The fact that the law has defined it as such, with a decision which causes every would-be suicide who has failed in his attempt, to be assigned to prison until he can be judged and sentenced for his guilty act, makes not the smallest difference on their conviction that they have a perfect right to die if they please. This fixed idea that their life has been given them as a prey, which they can either destroy or preserve as they think fit, prevails almost as widely among the upper classes—with whose idiosyncracies in this respect I propose to deal later—as it does amongst those who could not share the intellectual standpoint of their superiors. With them all reasoning on the subject simply amounts to this crude saying, "I did not ask to come alive, and I do not see that anyone has a right to say I am not to have done with it whenever I please." The fact that the law takes a different view of the criminality of such acts, brings under the notice of chaplains, prison visitors and others connected with our penal establishments, a variety of individual cases, some of which are simply astounding from the weakness or frivolous nature of the more immediate motives which have led men and women to plunge into the solemn mystery of death. While the two great influences I have mentioned—disbelief in God and a future state, or in self-murder as a crime, inevitably liable to punishment—remain with them ever as an underlying power to remove all restraint from their mind, the actual impulses which induce them to depart from the world, often at a moment's notice, are frequently so utterly inadequate, so almost ludicrous, that they seem to reveal in the uneducated mind a complete incapacity to estimate the comparative value of the present fleeting moment, and the long future laden with possible events, which they extinguish utterly for the sake of a desire accomplished that can affect only a few passing hours; for results that cannot under any circumstances endure more than the proverbial nine days' wonder, they will barter the fruitful heritage of a life that within the confines of this visible world only can give long years of useful work, of pleasant intercourse with others, of healthy effort and corresponding success in friendship and affection from congenial persons, or, to put the issues of existence at their lowest point, of physical or sensual enjoyment. To illustrate my meaning I will give the details of a most trifling and insignificant case, but effectual in its deadly purpose, which certainly sprang from the lowest range of human motive. A young girl, about nineteen years of age, sufficiently good looking to attract the notice of various young men in the neighbourhood, where she lived quite respectably and morally with her parents, seemed to find much pleasure in flirting with them all, while having no special affection for anyone in particular. She fluttered out and in amongst them like a butterfly, flying off to meet them in their evening resorts so soon as her day's work was done—now attracting, now repelling, pitting one against another, and accepting all their attentions and compliments which fed the conspicuous vanity that was her chief characteristic. But it happened after a time that another young woman came on the scene, infinitely more attractive than herself—in a better position in life, and superior to her in many ways that were instantly recognised by the young men who had hitherto been her followers. To descend to this humiliation was unbearable to her; she had been the principal person among that group for a long time, and she determined that she would be so again—for one day at least. She would arrange a lugubrious scene at which they should all be compelled to assist, and to speak her name with bated breath and sad recognition of an object of their admiration gone prematurely from the world. She went to her room and wrote a letter to her mother, in which she told her that her dead body would be brought home from the river next morning, and she charged her by all the love and indulgence she had ever shown her to carry out to the strictest letter the directions she now proceeded to give her. She was to fix the girl's funeral for the day and hour which would best accomplish the purpose that had sent her to her death. She was to write a separate letter of invitation to her burial, for each one of the young men who had been her admirers till her obnoxious rival appeared. She gave the name and address of each with the utmost care, and besought her mother, as a last act of kindness, to insist upon the appearance of every one of them at her interment, in terms which would make it impossible for them to refuse; and with that, for no other reason, she took leave of all her relations and of life itself. She placed the letter where her mother could not fail to find it, and then went out and most effectually drowned herself in the nearest river. Here, then, was this girl of blameless moral conduct, of good capacity for work and usefulness, possessed of sufficiently pleasant qualities to be an agreeable inmate of a happy home, who might have been a cherished wife and glad mother of well-loved children, who flung away the whole rich promise of her future to gratify, by anticipation only, the inordinate vanity which filled her whole being to an extent that drove from her thoughts all other considerations.

My object in making these researches into the hidden motives which goad helpless, unreasoning beings to their doom, is founded on the hope that I may be able, when I come to the close of my remarks, to suggest a remedy that may possibly—at least, to some slight degree—lessen the number of tragic occurrences of this nature, which darken with a touch of special gloom every page of our daily records. I trust, also, that my readers may take some interest in these investigations among the undiscovered depths of the human nature; for, according to the experts of certain sciences which deal with subliminal consciousness and duplex personality, we have much to learn respecting the mysterious problem of that existence which we all share alike.

